# Idiopathic mesenteric phlebosclerosis occurring in a patient with liver cirrhosis: A case report

**DOI:** 10.1097/MD.0000000000037608

**Published:** 2024-03-15

**Authors:** Congjie Zhang, Haijun Huang, Beibei Guo

**Affiliations:** a Department of Dermatology, Center for Plastic and Reconstructive Surgery, Zhejiang Provincial People’s Hospital (Affiliated People’s Hospital, Hangzhou Medical College), Hangzhou, Zhejiang, China; b Department of Infectious Diseases, Center for General Practice Medicine, Zhejiang Provincial People’s Hospital, People’s Hospital of Hangzhou Medical College, Hangzhou, Zhejiang, China; c Department of Gastroenterology, Center for General Practice Medicine, Zhejiang Provincial People’s Hospital, Affiliated People’s Hospital, Hangzhou Medical College, Hangzhou Zhejiang, China.

**Keywords:** case report, idiopathic mesenteric phlebosclerosis, liver cirrhosis

## Abstract

**Background:**

Idiopathic mesenteric phlebosclerosis (IMP) is a rare gastrointestinal disease with unclear etiology and pathogenesis. IMP occurring in a patient with liver cirrhosis is more scarcely reported than independent IMP. In this study, we reported a case of IMP occurring in a patient with liver cirrhosis, so as to provide a reference for understanding liver cirrhosis with IMP.

**Method:**

A 63-year-old man with liver cirrhosis was admitted in the hospital’s department of infectious disease because of fatigue and constipation for 1 month. The patient had an irregular medical history of antivirus drug and Chinese herbal medicine intake because of the hepatitis B virus infection. No other abnormalities were found in the functions of the liver, coagulation, renal, or complete blood count. Fecal occult blood tests were all positive in 5 detections. Contrast-enhanced computed tomography revealed liver cirrhosis and showed thickening of the wall of the right hemicolon and multiple calcifications of the mesenteric veins. Mesenteric vein computed tomography venography displayed diffuse colon mural thickening of the right colon and tortuous linear calcification line in the right colic veins. Colonoscopy revealed a purple-blue, swollen, rough, and vanished vascular texture mucosa. He was finically diagnosed as liver cirrhosis with IMP by a series of examinations during hospitalization.

**Results:**

His symptoms of fatigue and constipation subsided after conservative treatment and withdraw from Chinese herbal medicine. The patient experienced no obvious discomfort during the follow-up period.

**Conclusion:**

A comprehensive medical diagnosis is necessary for the discovery of IMP, especially IMP with liver cirrhosis. Liver cirrhosis maybe play a key role in the development of IMP. The regulatory mechanism of liver cirrhosis contributing to IMP needs to be further studied based on more clinical cases.

## 1. Introduction

Idiopathic mesenteric phlebosclerosis (IMP) is a rare gastrointestinal disease that causes ischemic colitis because of the noninflammatory, nonthrombotic stenosis of the mesenteric veins. IMP is commonly identified by characteristic multiple calcifications in the colonic wall and small mesenteric vessels with thickening of the right hemicolon in plain abdominal radiograph and computed tomography and is presented with purple-blue mucosa, colonic ulcerations, and edematous mucosa in colonoscopy.^[1,2]^ The etiology and pathogenesis of IMP remain poorly understood.

Chronic liver diseases are recognized as possible risk factors contributing to IMP. However, the association between IMP and chronic liver diseases is unclear.^[3]^ Few clinical cases with both IMP and chronic liver diseases have been reported among the rare IMP. Herein, we reported a case of IMP occurring in a patient with liver cirrhosis, so as to provide a reference for understanding liver cirrhosis with IMP. Readers can increase their understanding of the association between liver cirrhosis and IMP through this case and review of literatures.

## 2. Case report

We performed the anonymization of the patient in this study. The patient has provided informed consent for publication of the case. This study was reviewed and approved by the local ethics committee of Zhejiang Provincial People’s Hospital. The procedures were in accordance with the Helsinki Declaration of 1975, as revised in 2000.

**Figure 1.**
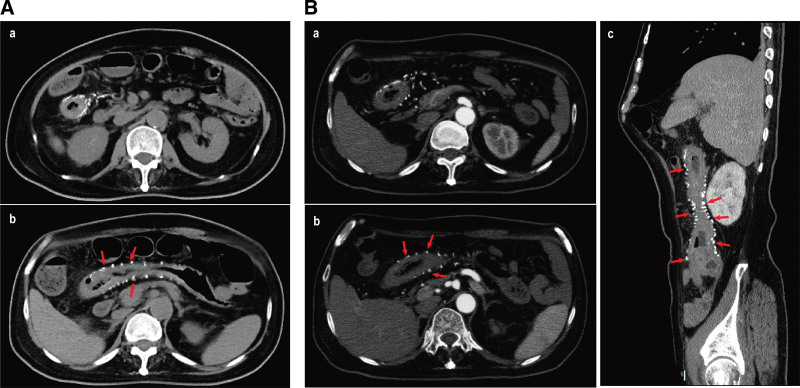
Abdominal CECT and mesenteric vein CTV of the patient with IMP and liver cirrhosis. (A) CECT-depicted thickening of the right hemicolon (A-a), and prominent calcifications of the mesenteric veins along the colonic wall (A-b). (B) CTV showed diffuse colon mural thickening from the ileocecal area to the hepatic flexure colon (B-a), and multiple calcifications were observed in the right colic veins (B-b and B-c). The arrow indicates calcifications. CECT = contrast-enhanced computed tomography, CTV = computed tomography venography, IMP = idiopathic mesenteric phlebosclerosis.

**Figure 2.**
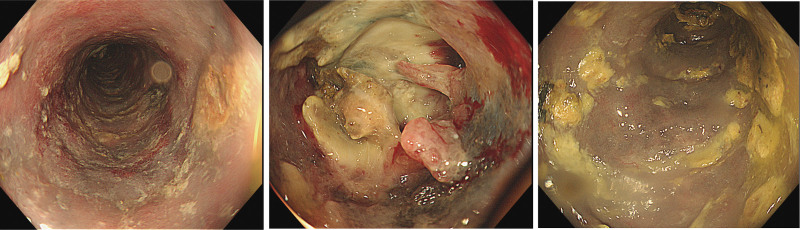
Colonoscopy of the patient with IMP and liver cirrhosis. Colonoscopy revealed a purple-blue, swollen, and rough mucosa, as well as annular mucosal eminence in the transverse colon. IMP = idiopathic mesenteric phlebosclerosis.

A 63-year-old man who was diagnosed with liver cirrhosis for the last 23 years was admitted to the hospital’s department of infectious disease because of fatigue and constipation for 1 month. He denied other discomfort in the form of abdominal pain, bloating, diarrhea, and so on. His past medical history included diabetes and hypertension. Medications included insulin, irbesartan, and hydrochlorothiazide to control blood glucose and pressure. Noteworthily, he had an irregular medical history of antivirus drug and Chinese herbal medicine intake because of the hepatitis B virus infection during the past 23-year period. The detailed ingredients and dosage of Chinese herbal medicines are untraceable. The patient also had a history of snake venom poisoning. His family and allergy history were unremarkable, and he had no history of smoking or drinking. His abdomen was soft and flat, and there was no tenderness in the physical examination. Spider nevi were found in the chest area. Laboratory tests were positive for HBsAg, HBeAb, and HBcAb and low levels of albumin (Alb 35.2 g/L, normal range: 40–55 g/L). No other abnormalities were found in the functions of the liver, coagulation, renal, or complete blood count. Anticytoplasmic antigen type 1 was positive in autoimmune liver disease antibody detection (1:34, normal range: 0–15). Tumor markers for liver cancer, including AFP, CEA, CA199, and ferritin, exhibited no abnormalities. Notably, fecal occult blood tests were all positive in 5 detections. Thus, abdominal ultrasound, contrast-enhanced computed tomography (CECT) and colonoscopy were performed. Abdominal ultrasound revealed that the right intestinal tube wall was thicker and there was no obvious ascites. CECT confirmed liver cirrhosis and showed thickening of the wall of the terminal ileum and ascending colon (Fig. 1A-a), prominent calcifications of the mesenteric veins along the colonic wall and blurred surrounding fat space (Fig. 1A-b), which indicated the occurrence of IMP. Mesenteric vein computed tomography venography showed diffuse colon mural thickening from the ileocecal area, and the ascending colon to the hepatic flexure colon (Fig. 1B-a), and multiple calcifications were observed in the right colic veins (Fig. 1B-b and B-c). Despite the absence of other abdominal discomfort than constipation, colonoscopy was performed because of a continuous positive fecal occult blood test and multiple calcifications on imaging of CECT and computed tomography venography. Colonoscopy revealed a purple-blue, swollen, rough, and vanished vascular texture mucosa along the intestinal wall extending from the sigmoid colon to the transverse colon. Furthermore, annular mucosal eminence was observed in the transverse colon. Colonoscopy was stopped at the transverse colon because of the patient’s intolerance (Fig. 2). Tissue sample from the annular mucosal eminence was collected for biopsy. Histological examinations detected inflammatory necrosis and partially proliferating granulation tissue. Overall, the patient with liver cirrhosis was diagnosed with IMP by imagological and endoscopic examinations. His symptoms of fatigue and constipation subsided after conservative treatment. The patient experienced no obvious discomfort during the follow-up period.

## 3. Discussion

IMP as a rare disease can be diagnosed by characteristic clinical manifestations, imaging findings, and endoscopic examination. IMP is identified as a noninflammatory and nonthrombotic chronic intestinal ischemia syndrome that exhibits classic multiple calcifications and thickening in the right hemicolon in imagological examination, purple-blue edematous mucosa and ulcerations on colonoscopy, and mucosal fibrosis and collagen deposition on histology.^[2,4]^ IMP has gradual onset and progression, in which chronic ischemia of the colon could be attributed to disturbed venous return because of the calcification of the mesenteric venous system.^[5]^ The case reports in Asia, especially Japan and China, are obviously higher than those in Europe and the United States. Previous studies in English language suggest that IMP tends to occur in females.^[3,4,6]^ However, more males with IMP are reported than females in Chinese patients. Ding et al^[7]^ reported 10 cases who were all males admitted in their hospital from January 2013 to May 2019. All 8 patients were also male in the case report of Wen et al.^[5]^ Chen et al^[8]^ reported that 23 of 25 IMP patients were male. The limited number of patients in case reports may be insufficient to show a trend of sex difference so far. There may also exist an association with the region-specific lifestyle or culture in the incidence of IMP.

The etiology and pathogenesis of IMP are still unknown. Many risk factors are hypothesized to contribute to the development of IMP. Chronic diseases were recognized as important risk factors of IMP, including cardiovascular disease, chronic renal disease, cancer, chronic liver disease, diabetes mellitus, hyperlipidemia, rheumatism, schistosomiasis, and so on.^[3]^ Chronic diseases are commonly accompanied by a slow hemodynamic change for a long time. The mesenteric veins with slow blood flow may gradually be calcified under abnormal shear stress. Otherwise, many cases show that long-term intake of Chinese herbs may play a key role in the pathogenesis of IMP.^[5,9–11]^ Miyazaki et al^[2]^ reported that IMP occurred in a wife and her husband, who both had been taking a large amount of the herbs for a long time. The authors concluded that IMP may be associated with herbal medicine intake and lifestyle. Herbal medicines are generally used in chronic renal disease, cancer, and chronic liver disease in Asian countries. The components of herbal medicines are complicated, and the metabolism and pharmacokinetics of many components are not fully understood. The long-term safety of herbal medicine needs to be further explored and elucidated. Aristolochic acid causes vascular injury in herb-induced nephropathy, which later results in certain ischemia-induced alterations and interstitial fibrosis.^[12]^ Even similar to herb-induced nephropathy also may contribute to the development of IMP. Moreover, geniposide (called *sansisi* in Chinese herbs) was commonly used for chronic liver and renal disease by reducing oxidative stress and suppressing inflammation.^[13,14]^ By analyzing the medicine history of many patients, studies have concluded that geniposide plays a key role in IMP.^[5,9]^ However, few study reports on the mechanism of geniposide contributing to IMP. Further investigations are required to clarify the possible role of Chinese herbs in the pathogenesis of IMP.

IMP may present as symptoms of ischemic colitis, including abdominal pain, distension hematochezia, intestinal obstruction, etc, or without any abdominal discomfort at all. The primary diagnosis of IMP is usually based on the thickening of the colonic wall and typical calcification of the mesenteric veins in imaging findings and the purple-blue or dark purple mucosa in colonoscopy.^[15]^ Otherwise, colonoscopy may also reveal colonic ulcerations and edematous mucosa in some patients. The gold criterion of IMP is histopathologic findings presented with submucosal fibrosis and increased collagen deposition. The clinical symptoms and laboratory and physical examination of IMP are not typical, so it is easy to miss diagnoses and give a misdiagnosis.^[16]^ The differential diagnosis needs to be emphasized between IMP and the following diseases: Ischemic colitis made from atherosclerosis, patchy calcification is generally in the larger branches of the mesenteric artery, which is different from multiple calcifications of IMP;^[17,18]^ Collagenous colitis with a thickening of the collagen layer in the lining of the colon; its pathology shows the deposited colloidal particles in bands below the epithelial cells and chronic inflammation of the colon,^[19,20]^ Chronic intestinal schistosomiasis with a wide range of calcifications; the submucosa becomes thickened with fibrous tissue containing massive amounts of calcified eggs rather than mesenteric vein calcification,^[21]^ Melanosis coli with characterization by a black or brown mucosa in colonoscopy, a reversible condition influenced by living habits, bowel function and anthraquinone drugs.^[22]^ It is important to withdraw herbal medicine for IMP patients. Conservative treatment is commonly administered, and the prognosis of IMP is benign. However, surgery may be considered in patients with persistent symptoms or bowel obstruction.

Only a few cases of IMP complicated by chronic diseases have been reported heretofore, requiring a larger data set to identify the association between IMP and concomitant diseases. Chronic liver disease and Chinese herbal medicine intake can both contribute to the development of IMP in the reported literature.^[3,7]^ In our case, the patient was admitted in the hospital’s department of infectious disease because of liver cirrhosis for the last 23 years and was then diagnosed with IMP by a series of examinations during hospitalization. The patient had an irregular medicine history of antivirus drug and Chinese herbal medicine intake owing to the hepatitis B virus infection. Liver cirrhosis and Chinese herbal medicine intake exist simultaneously in our case. Two risk factors contribute to the incidence of IMP, though the mechanism remains unknown. In a previous study, portal hypertension and liver dysfunction due to liver cirrhosis were reported in the case of IMP.^[23]^ In our case, portal hypertension and liver decompensation were excluded by clinical manifestations and examination results, which may indicate that there is another way to influence IMP in chronic liver disease. Though the right hemicolon was not observed in colonoscopy because of the patient’s intolerance, there were classic characterizations, including a purple-blue mucosa from the sigmoid colon to the transverse colon, thickening of the wall of the right hemicolon and calcifications of the mesenteric veins. No mucosal fibrosis and collagen deposition were detected on histology because of the biopsy tissue collected from annular mucosal eminence. Overall, the diagnosis of IMP was confirmed by characteristic imaging and endoscopic findings in our case.

## 4. Conclusion

A comprehensive medical diagnosis is necessary for the discovery of IMP, especially IMP with liver cirrhosis. Liver cirrhosis maybe play a key role in the development of IMP. The regulatory mechanism of liver cirrhosis contributing to IMP needs to be further studied based on more clinical cases.

## Author contributions

**Data curation:** Congjie Zhang.

**Funding acquisition:** Haijun Huang.

**Investigation:** Congjie Zhang.

**Supervision:** Haijun Huang.

**Writing – original draft:** Congjie Zhang, Beibei Guo.

**Writing – review & editing:** Congjie Zhang, Beibei Guo.

## References

[1] Siao D Thoeni R Grenert JP. A rare presentation of abdominal pain: idiopathic mesenteric phlebosclerosis. Am J Gastroenterol. 2012;107:1759–60. 23160302 10.1038/ajg.2012.289

[2] Miyazaki M Nakamura S Matsumoto T. Idiopathic mesenteric phlebosclerosis occurring in a wife and her husband. Clin Gastroenterol Hepatol. 2009;7:e32–3. 19514111 10.1016/j.cgh.2009.01.024

[3] Oshitani N Matsumura Y Kono M. Asymptomatic chronic intestinal ischemia caused by idiopathic phlebosclerosis of mesenteric vein. Dig Dis Sci. 2002;47:2711–4. 12498290 10.1023/a:1021090113274

[4] Iwashita A Yao T Schlemper RJ. Mesenteric phlebosclerosis: a new disease entity causing ischemic colitis. Dis Colon Rectum. 2003;46:209–20. 12576895 10.1097/01.DCR.0000044720.43258.6E

[5] Wen Y Chen YW Meng AH. Idiopathic mesenteric phlebosclerosis associated with long-term oral intake of geniposide. World J Gastroenterol. 2021;27:3097–108. 34168411 10.3748/wjg.v27.i22.3097 PMC8192294

[6] Shibata H Nishikawa J Sakaida I. Dark purple-colored colon: sign of idiopathic mesenteric phlebosclerosis. Dig Endosc. 2014;26:604–5. 10.1111/den.12299 24720619

[7] Ding J Zhang W Wang L. Idiopathic mesenteric phlebosclerosis: clinical and CT imaging characteristics. Quant Imaging Med Surg. 2021;11:763–71. 33532275 10.21037/qims-20-301 PMC7779937

[8] Chen W Zhu H Chen H. Phlebosclerotic colitis: our clinical experience of 25 patients in China. Medicine (Baltimore). 2018;97:e12824. 30412073 10.1097/MD.0000000000012824 PMC6221691

[9] Hiramatsu K Sakata H Horita Y. Mesenteric phlebosclerosis associated with long-term oral intake of geniposide, an ingredient of herbal medicine. Aliment Pharmacol Ther. 2012;36:575–86. 22817400 10.1111/j.1365-2036.2012.05221.x

[10] Shimizu S Kobayashi T Tomioka H. Involvement of herbal medicine as a cause of mesenteric phlebosclerosis: results from a large-scale nationwide survey. J Gastroenterol. 2017;52:308–14. 27220772 10.1007/s00535-016-1218-9

[11] Nishiura H Nakase H Chiba T. Sustained abdominal discomfort in a 57-year-old woman. Idiopathic mesenteric phlebosclerosis. Gut. 2010;59:578–94. 20427391 10.1136/gut.2009.189647

[12] Vanherweghem JL Depierreux M Tielemans C. Rapidly progressive interstitial renal fibrosis in young women: association with slimming regimen including Chinese herbs. Lancet. 1993;341:387–91. 8094166 10.1016/0140-6736(93)92984-2

[13] Chen XY Jiang WW Liu YL. Anti-inflammatory action of geniposide promotes wound healing in diabetic rats. Pharm Biol. 2022;60:294–9. 35130118 10.1080/13880209.2022.2030760 PMC8823683

[14] Dusabimana T Park EJ Je J. Geniposide improves diabetic nephropathy by enhancing ULK1-mediated autophagy and reducing oxidative stress through AMPK activation. Int J Mol Sci. 2021;22:1651. 33562139 10.3390/ijms22041651 PMC7915505

[15] Tong T Fu J Kong Y. Recurrent abdominal pain in a 61-year-old woman. Gastroenterology. 2023;164:887–90. 36343673 10.1053/j.gastro.2022.10.032

[16] Guo F Zhou YF Zhang F. Idiopathic mesenteric phlebosclerosis associated with long-term use of medical liquor: two case reports and literature review. World J Gastroenterol. 2014;20:5561–6. 24833888 10.3748/wjg.v20.i18.5561 PMC4017073

[17] Dimitrijević I Micev M Saranović D. Ischaemic colitis – review. Acta Chir Iugosl. 2008;55:89–95. 19069699 10.2298/aci0803089d

[18] Trotter JM Hunt L Peter MB. Ischaemic colitis. BMJ. 2016;355:ii6600. 10.1136/bmj.i6600 28007701

[19] Hatemi AI Senateş E Dobrucali A. Collagenous colitis: a retrospective survey of patients with chronic diarrhea. Hepatogastroenterology. 2011;58:1963–7. 22024068 10.5754/hge11228

[20] Jobse P Flens MJ Loffeld RJ. Collagenous colitis: description of a single centre series of 83 patients. Eur J Intern Med. 2009;20:499–502. 19712853 10.1016/j.ejim.2009.03.004

[21] Olveda DU Olveda RM McManus DP. The chronic enteropathogenic disease schistosomiasis. Int J Infect Dis. 2014;28:193–203. 25250908 10.1016/j.ijid.2014.07.009

[22] Yang N Ruan M Jin S. Melanosis coli: a comprehensive review. Gastroenterol Hepatol. 2020;43:266–72. 32094046 10.1016/j.gastrohep.2020.01.002

[23] Kitamura T Kubo M Nakanishi T. Phlebosclerosis of the colon with positive anti-centromere antibody. Intern Med. 1999;38:416–21. 10397079 10.2169/internalmedicine.38.416

